# The chromosomes of Tsing-Ling pika, *Ochotona huangensis* Matschie, 1908 (Lagomorpha, Ochotonidae)

**DOI:** 10.3897/CompCytogen.v6i4.3183

**Published:** 2012-10-08

**Authors:** Alexey A. Vakurin, Vladimir P. Korablev, Jiang Xue-Long, Tat'iana V. Grigor'eva

**Affiliations:** 1 Institute of Biology and Soil Science, Far Eastern Branch of the Russian Academy of Sciences; pr. Stoletiya 159, Vladivostok, 690022, Russia; 2 Kunming Institute of Zoology, the Chinese Academy of Sciences; Kunming, Yunnan, 650223, China; 3 Faculty of Biology, M. V. Lomonosov Moscow State University; Vorob’evy Gory 1, p. 12, Moscow, 119991, Russia

**Keywords:** *Ochotona huangensis*, pika, karyotype, chromosome, C-banding

## Abstract

The karyotype of the Tsing-Ling (Huanghe) pika, *Ochotona huangensis* Matschie, 1908 from the forest habitats of the Qinling Mountains (Shaanxi Province, China) was described for the first time. The chromosome set contains 42 chromosomes (NFa=80). The autosomes are 15 meta-submetacentric pairs and 5 subtelocentric pairs. The X chromosome is a medium sized submetacentric; the Y chromosome is a small sized acrocentric. C-banding revealed a localization of heterochromatin in the pericentromeric regions of all autosomes.

## Introduction

The pikas *Ochotona* Link, 1795 are small (12–28 cm long) mammals of the order Lagomorpha Brandt, 1855. The developed sound signaling is a characteristic feature of most northern *Ochotona* species. They live either alone or in colonies, preferring taluses or open plains. The pikas find refuges in the crevices between rocks or dig burrows (Sokolov et al. 1994, Hoffmann and Smith 2005).

These animals occur in North America from Alaska to New Mexico. In the Old World pikas are distributed from the Arctic coast to the northern regions of Iran, Afghanistan, Pakistan, India and Burma, from the Polar Urals in the West to the Pacific coast in the East, including Chukotka, Kamchatka peninsula, Hokkaido Island and also in territory of North Korea (Sokolov et al. 1994, Hoffmann and Smith 2005).

The pikas are one of the most ancient groups of the placental mammals (Gureev 1964, Ivanitskaya 1989, Lopatin and Aver’yanov 2008, Rose et al. 2008). The morphological criteria of species diagnosis were ascertained for many described *Ochotona* species. The pikas have rather distinct interspecific differentiation of karyotypes (2n=38-68) that helps to solve controversial taxonomic issues. Most species have stable karyotypes without geographic variability and intrapopulation polymorphism (Ivanitskaya 1989). However, two chromosomal forms of uncertain taxonomic rank were revealed for * alpina* group (Formozov et al. 2006).

The majority of modern taxonomists recognize 30 species of pikas and they divide them into three subgenera: *Pika* Lacepede, 1799, *Ochotona* Link, 1795 and *Conothoa* Lyon, 1904 (Hoffmann and Smith 2005). The karyotypes at least of 17 pika species were described (Table 1). These species are mainly from northern and temperate latitudes. Information on the differential staining of chromosomes is available for 13 species. Comparative analysis of G-banding pika chromosomes showed a high degree of similarity between the karyotypes of several species: *Ochotona alpina* – *Ochotona pallasi*, *Ochotona pusilla* – *Ochotona princeps*, *Ochotona rutila* – *Ochotona rufescens* (Ivanitskaya 1991).

**Table 1.** Subgenera system of the genus *Ochotona* and variability of the diploid chromosome number (2n). NF – the fundamental number of chromosomal arms.

Subgenus	Species	2n	NF	Banding methods	References
*Pika*	*Ochotona argentata* Howell, 1928	38	76	C, NOR	Formozov et al. 2004
	*Ochotona hoffmanni* Formozov et al., 1996	38	76	G, C	Formozov and Baklushinskaya 1999
	*Ochotona pallasi* (=*pricei*) Gray, 1867	38	–	–	
			76	G, C	Ivanitskaya 1991
	*Ochotona hyperborea* Pallas, 1811	40	–	–	Hayata and Shimba 1969
			–	–	Vorontsov and Ivanitskaya 1973
			76	C	Ivanitskaya 1991
	*Ochotona alpina* Pallas, 1773	42	–	–	Vorontsov and Ivanitskaya 1973
			78	G, C	Ivanitskaya 1991
			–	–	Formozov et al. 2006
	*Ochotona collaris* Nelson, 1893	68	90	–	Rausch and Ritter 1973
	*Ochotona princeps* Richardson, 1828	68	86	–	Adams 1971
				G, C	Stock 1976
*Ochotona*	*Ochotona huangensis* Matschie, 1908	42	84	C	Our data
	*Ochotona curzoniae* Hodgson, 1858	46	68	G, C	Tan and Bai 1987
	*Ochotona nubrica* Thomas, 1922	48	–	–	Formozov et al. (personal communication)
	*Ochotona dauurica* Pallas, 1776	50	–	–	Vorontsov and Ivanitskaya 1973
			72	G, C	Ivanitskaya 1991
	*Ochotona pusilla* Pallas, 1769	68	–	–	Vorontsov and Ivanitskaya 1973
			106	G, C	Ivanitskaya 1991
*Conothoa*	*Ochotona forresti* Thomas, 1923	54	–	DAPI	Ye et al. 2011
	*Ochotona rufescens* Gray, 1842	60	86	–	Nadler et al. 1969
			–	–	Vorontsov and Ivanitskaya 1973
			–	G, C	Kimura et al. 1983
			90	G, C	Ivanitskaya 1991
	*Ochotona roylei* Ogilby, 1839	62	–	G, NOR	Capanna et al. 1991
	*Ochotona macrotis* Gunther, 1875	62	86	–	Vorontsov and Ivanitskaya 1973
	*Ochotona rutila* Severtsov, 1873	62	–	–	Vorontsov and Ivanitskaya 1973
			86	G, C	Ivanitskaya 1991
	*Ochotona ladacensis* Gunther, 1875	68	–	–	Formozov et al. (personal communication)

Up to 24 species of pika inhabit China (Wang 2003), but the karyotypes of only five species have been described for this territory: *Ochotona curzoniae* Hodgson, 1858 (Tan and Bai 1987), *Ochotona ladacensis* Gunther, 1875, *Ochotona nubrica* Thomas, 1922 (Formozov et al. personal communication), *Ochotona argentata* Howell, 1928 (Formozov et al. 2004), *Ochotona forresti* Thomas, 1923 (Ye et al. 2011).

During the last four decades, the systematics of the northern Palearctic and North American pikas has been well developed, but the system of subgenera and superspecies groups was periodically reconsidered with increase of number of morphological, morpho-ecological features and descriptions of karyotypes (Ivanitskaya 1991). Later it was corrected by multiple molecular data (Yu et al. 2000, Niu et al. 2004, Formozov et al. 2006, Lissovsky et al. 2007, Lanier and Olson 2009).

In this paper the karyotype of *Ochotona huangensis* Matschie, 1908 is described for the first time. This species has a few synonyms of common names: Tsing-Ling pika, Huanghe pika, Qinling pika. We will use the common name as Tsing-Ling pika, before conducting the full revision of this species. We adhered to intrageneric taxonomy proposed by Hoffmann and Smith (2005), in which *Ochotona huangensis* belongs to the subgenus *Ochotona*. A level of variation of the diploid chromosome numbers in subgeneric groups of the genus *Ochotona* is discussed on the basis of our own and literature data.

## Material and methods

One male of *Ochotona huangensis* was used as a material for this study. It was caught on Sept. 12, 2005 during the joint Russian-Chinese expedition to the Qinling Mountains near the Foping village of Shaanxi Province, China. The pika was caught on a glade of the pine-oak forest, at height less than 1800 m (33°28'36,3"N, 108°30'18,6"E). This was slightly below the typical habitat for the Tsing-Ling (Huanghe) pika: a birch-fir forest located above 2000 m (Qin et al. 2007). This specimen is stored under the № 0509391 in the museum of Kunming Institute of Zoology. The karyotype of one male of *Ochotona dauurica* Pallas, 1776 was studied for comparison. The Daurian pika was caught in 2004 near the Tsagan-Oluy village (50°30'N, 117°3'25"E) of Borzya Distr. Transbaikalia, Russia.

Identification of the pika from the Qinling Mountains was performed by morphological characters. We used a molecular express analysis of the cytochrome *b* gene of mtDNA for confirming of taxonomic status of this specimen to the species *Ochotona huangensis*. Total genomic DNA was extracted from liver tissue by standard protocol (Arrighi et al. 1968). We used a standard polymerase chain reaction (PCR) for full-length sequences cytochrome *b* gene (1140 bp) amplification with specially designed primers:

L14075och 5’ – gta tgt cat aat tct tac atg ga – 3’

H15374och 5’ – gta agc cga ggg cgt ctt tg – 3’

The primers were designed according to published whole mitochondrial sequence of pika *Ochotona collaris* (GenBank NCBI (www.ncbi.nlm.nih.gov ) № NC_003033). The PCR program consisted of 94 °C for 5 min followed by 35 cycles at 94 °C for 1 min, 62 °C for 1 min, and 72 °C for 3 min. A final amplification step completed the PCR at 72 °C for 7 min.

The PCR products were purified by Sin Column PCR Product Purification Kit (Evrogen, Moscow, Russia). The directly sequencing of the purified PCR products was performed using ABI PRISM BigDye^TM^ Terminator v3.1 (Applied Biosystems, Inc., Foster City, California) with an automatic DNA sequencer (Model ABI PRISM 3100-Avant Genetic Analyzer; Applied Biosystems, Inc., Foster City, California). The same primers were used for sequencing PCR from both directions.

**Figure 1.**
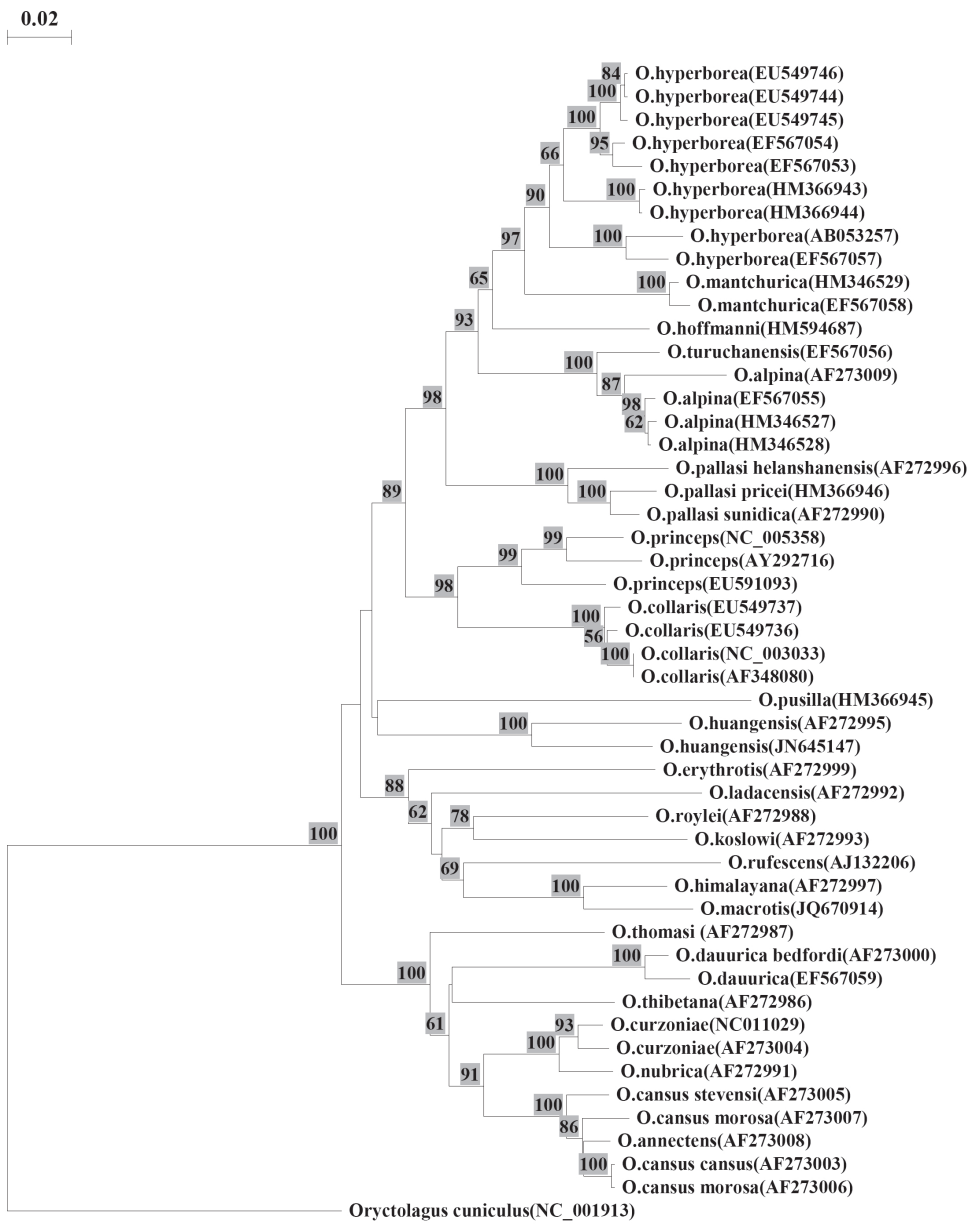
Neighbor-joining distance tree constructed using the Kimura two-parameter model for complete sequence cytochrome b (1140 bp). Numbers on branches indicate bootstrap support; values less than 50 are not shown. Numbers following the species names indicate the GenBank accession numbers.

The obtained sequence (GenBank NCBI № JN645147) was compared with full-length cytochrome *b* (1140bp) of 23 pikas species published by different authors in GenBank. The alignment of sequences was conducted by the program BIOEDIT v7.0.9 (Tom Hall, Ibis Biosciences). The genetic distances were estimated with neighbor-joining method, using Kimura two-parameter model. The tree was constructed by including all transitions and transversions with TREECON v3.1b (Yves Van De Peer, Germany). A rabbit *Oryctolagus cuniculus* Linnaeus, 1758 was selected as an outgroup (Fig. 1).

Method of cell division stimulation in the red bone marrow with baker’s yeast solution was used for preparation of chromosomal slides (Lee and Elder 1980). The slides were made by standard method (Ford and Hamerton 1956). The procedure of differential staining (C-banding) was held for detection of structural heterochromatin (Sumner 1972).

The chromosomal slides were analyzed on light microscope AxioSkop 40 with lens x100. Photographs were performed with the digital camera AxioCamHR using the program AXIOVISION 4.7 (Carl Zeiss MicroImaging GmbH, Germany). The morphology of the chromosomes was assessed visually without measurements (Orlov 1974).

## Results and discussion

Independence ofthe *Ochotona huangensis* taxon was suggested by molecular studies (Yu et al. 2000, Niu et al. 2004), but the morphological revision of specimens used in these articles was never done, so we can stick to only one fact. Our data of the molecular express analysis showed that the sequence of our specimen had maximum similarity to that sequence of specimen which was identified as *Ochotona huangensis* by Yu (2000) (Fig. 1).

**Figure 2.**
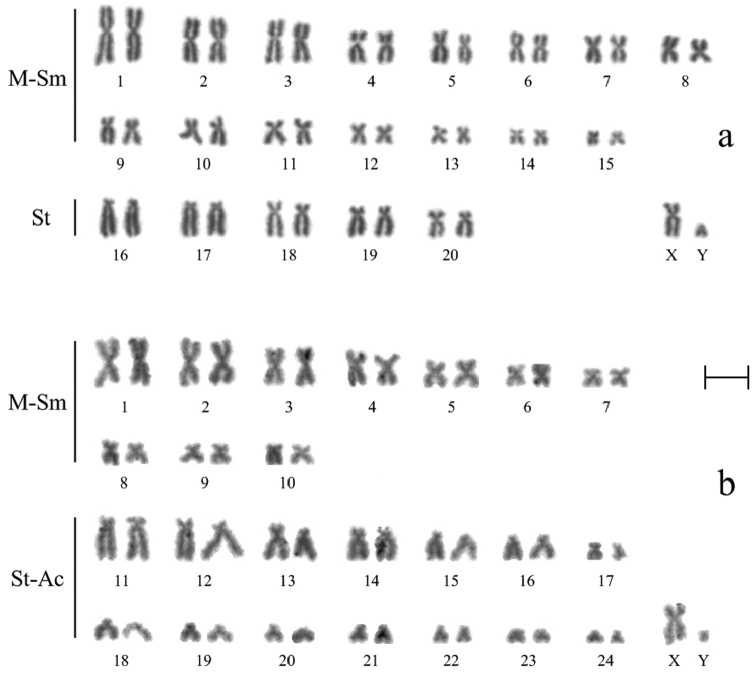
Routine stained karyotypes of *Ochotona huangensis* (**a**) and *Ochotona dauurica* (**b**): **M-Sm** meta-submetacentric chromosomes, **St** subtelocentric chromosomes, **St-Ac** subtelo- and acrocentric chromosomes. Bar = 5 μm.

According to the results of counting on 40 metaphase plates, the diploid chromosome number of *Ochotona huangensis* is 42 (NFa=80). Morphologically two groups of autosomes were identified. The first group consists of 15 pairs (3 large, 8 medium and 4 small) meta-submetacentric chromosomes. The second group consists of 5 pairs rather large, gradually decreasing in size, subtelocentric chromosomes. The X chromosome is a medium sized submetacentric, the Y chromosome is a small acrocentric (Fig. 2a).

**Figure 3.**
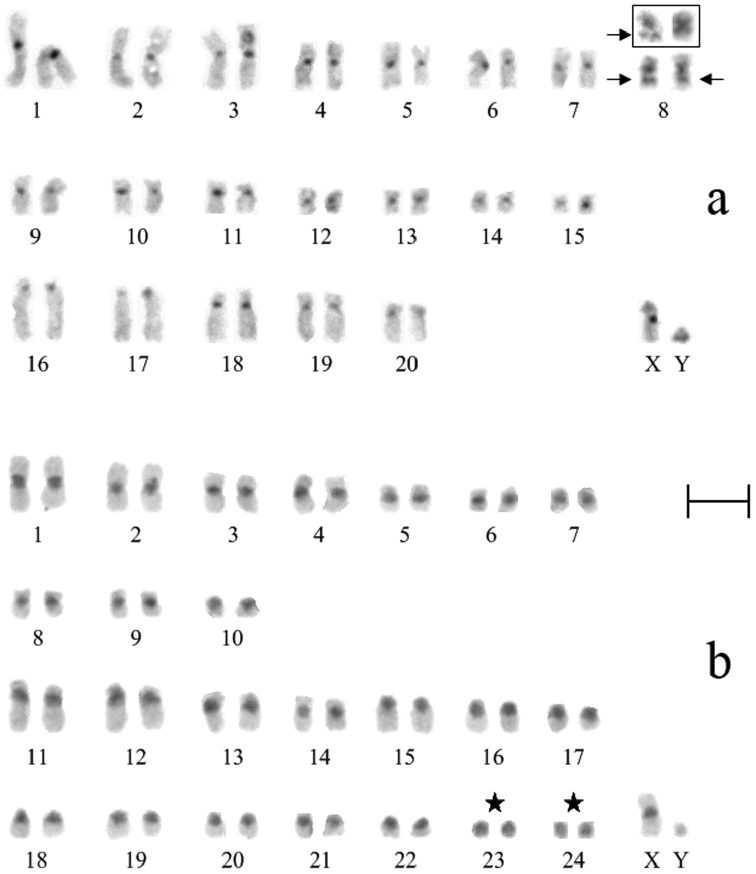
C-banded karyotypes of *Ochotona huangensis* (**a**) and *Ochotona dauurica* (**b**): ★ – intercalary heterochromatic blocks, → – autosomes entirely consisted of heterochromatin. Bar = 5 μm.

**Figure 4.**
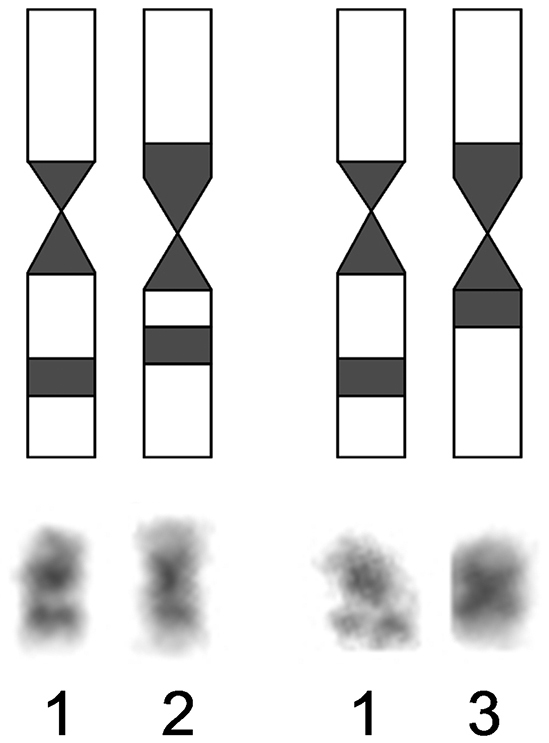
Scheme of localization of heterochromatic blocks on 8-th pair of *Ochotona huangensis*: 1 – the first homologue, 2 and 3 – the second homologue in two variants.

Nineteen metaphase cells stained for the structural heterochromatin (C-banding) were analyzed. The clearly stained pericentromeric heterochromatic blocks, which sizes were approximately the same, were identified at all chromosomes of *Ochotona huangensis*. The heteromorphism was detected by localization of heterochromatic blocks on the 8-th pair of autosomes. An intercalary heterochromatic block was always detected in the long arm of one homologue of the 8-th pair. Also, that homologue had the pericentromeric block of heterochromatin. In the second homologue of this pair, the intercalary heterochromatic block was detected in nine metaphase cells. In the remaining cells, only the larger pericentromeric heterochromatic block was detected in this homologue. By that, the euchromatic site, which separates the intercalary heterochromatic block, was broader on the first homologue than that on the second homologue (Fig. 4). We can’t characterize this phenomenon in details and discuss about its nature, because of the absence of sufficient material. So we leave it only as an observed fact. The X chromosome has a pericentromeric block of heterochromatin. The heterochromatic region occupies 2/3 of the lower arm on the Y chromosome (Fig. 3a).

The Daurian pika, which like *Ochotona huangensis* belongs to the subgenus *Ochotona*, was studied for a comparative karyotype analysis. The karyotype of *Ochotona dauurica* contains 50 chromosomes (NFa=68) which are grouped in 10 meta-submetacentric pairs (3 large, 2 medium and 5 small) and 14 subtelo- and acrocentric pairs of autosomes. The X chromosome is a submetacentric, similar in size to the 3-rd or 4-th pairs of autosomes, the Y chromosome is a very small acrocentric (Fig. 2b). The karyotype of the Daurian pika does not differ from that which was previously described in the literature (Vorontsov and Ivanitskaya 1969, 1973).

Analysis of 15 C-stained metaphase plates showed that all autosomes of *Ochotona dauurica* have the large pericentromeric heterochromatic blocks which were intensively stained. The 10-th and 15-th – 22-th pairs of autosomes have completely heterochromatic short arms. The last two small pairs of autosomes (23-th and 24-th) are composed of heterochromatin entirely. The large pericentromeric block of the X chromosome occupies 1/3 of the long arm. Heterochromatic structure of the Y chromosome was not confirmed (Fig. 3b) compared with published data (Ivanitskaya 1991).

An obvious resemblance between the karyotypes of *Ochotona huangensis* and *Ochotona dauurica* was seen by the routine staining, despite of some bigger size of the first pair of *Ochotona huangensis*. The first four meta-submetacentric pairs of *Ochotona dauurica* are similar to the 2nd – 5-th pairs of *Ochotona huangensis* autosomes. The remaining five meta-submetacentric pairs of *Ochotona dauurica*, except the 10-th pair, are similar to the last five pairs of the first group of *Ochotona huangensis* autosomes. The 11-th – 15-th autosomes of *Ochotona dauurica* are very similar to the second subtelocentric group of *Ochotona huangensis* by morphology and sizes, with a loss of the little part of the upper arm on the 20-th pair. The absence of G-stained chromosomes not allows us to do unambiguous conclusion about the relationship between the karyotypes of *Ochotona dauurica* and *Ochotona huangensis*. Such species as *Ochotona alpina* (2n=42), *Ochotona hyperborea* (2n=40), *Ochotona pallasi* (2n=38) and *Ochotona argentata* (2n=38) of the subgenus *Pika* (Vorontsov and Ivanitskaya 1973, Ivanitskaya 1991) are close to *Ochotona huangensis* by the diploid chromosome number. However, they have more significant differences in relation of morphological groups and sizes of chromosomes.

The C-banding patterns of *Ochotona dauurica* specimens from Transbaikalia (near the station Armagotuy) and Mongolia (Selenge aimag, near Shamar) (Ivanitskaya 1991) differs slightly from the specimen studied by us. Four pairs of subtelo-acrocentric autosomes have euchromatic material on the short arms in our pika. According to the data obtained by Ivanitskaya (1991), euchromatic material was on the short arms only on one pair. This pair is the largest and it corresponds to our 11-th pair. In addition, Ivanitskaya (1991) described three completely heterochromatic pairs, but according to our data, only last two pairs of autosomes have such features. These differences may be due to interpopulation variability as well as influence of different C-staining procedures of chromosomal slides. However, the reason of these differences remains unclear, because of the absence of sufficient material at present.

A tendency of heterochromatin decreasing is confirmed in row of pikas: from species with a large number of chromosomes to species with a smaller number, while comparing the overall C-banding pattern of *Ochotona dauurica* and *Ochotona huangensis* (Formozov et al. 2004). Perhaps, this indicates a loss of the heterochromatic material as a result of the chromosomal rearrangements.

The species *Ochotona alpina* (subgenus *Pika*) is similar to *Ochotona huangensis* by the diploid chromosome number, but it has another arrangement of heterochromatin. Pericentromeric heterochromatin is detected only on 6 submetacentric and 5 subtelocentric pairs of *Ochotona alpina* autosomes (Ivanitskaya 1991). Four submetacentric pairs of *Ochotona alpina* (especially the first pair) have the larger heterochromatic blocks than the corresponding pairs of *Ochotona huangensis*. Two large subtelocentric pairs of *Ochotona alpina* also have the larger blocks of heterochromatin in comparison with the subtelocentric pairs of *Ochotona huangensis*. The remaining three minor subtelocentric pairs of *Ochotona alpina*, which contain the pericentromeric heterochromatin, have no analogues in the karyotype of *Ochotona huangensis*. Besides, the X chromosome of *Ochotona alpina* has no heterochromatin unlike *Ochotona huangensis*. The Y chromosome of *Ochotona alpina* is composed of heterochromatin entirely (Ivanitskaya 1991).

The molecular studies of the genus *Ochotona* (Yu et al. 2000, Formozov et al. personal communication) showed division of pikas for three superspecies groups: 1. *Pika* – northern pikas and Mongolian pika; 2. *Ochotona* – shrub-steppe pikas except Mongolian, Ladak and Kozlov’s pikas; 3. *Conothoa* – mountain pikas with Ladak and Kozlov’s pikas. At present, the statuses of subgenera are given for these groups of pikas (Hoffmann and Smith 2005). Formozov et al. (personal communication) suggested the existence of variation of the diploid chromosome number for each subgenus (group) of pikas. The karyotypes of the subgenus *Pika* species have 38-42 chromosomes. The species of the subgenus *Ochotona* have the karyotypes with 46-50 chromosomes. The pikas of the subgenus *Conothoa* have 60-62 chromosomes in the karyotypes. Moreover, there are species with 2n=68 in each subgenus.

The position of *Ochotona huangensis* is ambiguous in this system. According to the data of study of the cytochrome *b* and the ND4 gene (Yu et al. 2000), *Ochotona huangensis* is very far distant from the group of shrub-steppe pikas. Also, *Ochotona huangensis* is allocated to a separate independent group by analysis of the cytochrome *b* of 27 pikas species (Niu et al. 2004). At present, *Ochotona huangensis* (2n=42) belongs to the subgenus *Ochotona* (Hoffmann and Smith 2005). If the view point of Hoffmann and Smith is true, our data extend the level of variation of the diploid chromosome number for the subgenus *Ochotona*. In this case, there is no border with the subgenus *Pika* by this indicator. Thus, *Ochotona huangensis* is significantly diverging from the main group of the subgenus *Ochotona* by main karyotypic characteristics that corresponds to the data of mtDNA study (Yu et al. 2000, Niu et al. 2004). The recent cytogenetic study of *Ochotona forresti* (2n=54) (Ye et al. 2011) also greatly expands karyotypic variability of the subgenus *Conothoa*. As yet, the karyotypes of eight species of the subgenus *Conothoa* and three species of the subgenus *Ochotona* are not investigated. It is not excluded that the new karyotypic data will changed the level of the diploid numbers variation between all subgenera of the genus. Thus, we assume that the karyotypic system of the genus *Ochotona* can not be constructed completely without studying cytogenetic characteristics of all species of pikas.
